# Micro-heterogeneity of malaria transmission in the Peruvian Amazon: a baseline assessment underlying a population-based cohort study

**DOI:** 10.1186/s12936-017-1957-y

**Published:** 2017-08-04

**Authors:** Angel Rosas-Aguirre, Mitchel Guzman-Guzman, Dionicia Gamboa, Raul Chuquiyauri, Roberson Ramirez, Paulo Manrique, Gabriel Carrasco-Escobar, Carmen Puemape, Alejandro Llanos-Cuentas, Joseph M. Vinetz

**Affiliations:** 1 Instituto de Medicina Tropical Alexander von Humboldt, Universidad Peruana Cayetano Heredia, Lima 31, Peru; 2 Research Institute of Health and Society (IRSS), Université Catholique de Louvain, 1200 Brussels, Belgium; 3 Laboratorio ICEMR-Amazonia, Laboratorios de Investigación y Desarrollo, Facultad de Ciencias y Filosofia, Universidad Peruana Cayetano Heredia, Lima 31, Peru; 4 Departamento de Ciencias Celulares y Moleculares, Facultad de Ciencias y Filosofia, Universidad Peruana Cayetano Heredia, Lima 31, Peru; 5 Facultad de Salud Pública y Administración, Universidad Peruana Cayetano Heredia, Lima 31, Peru; 6 Division of Infectious Diseases, Department of Medicine, University of California San Diego School of Medicine, 9500 Gilman Drive MC0760, Biomedical Research Facility-2, Room 4A16, La Jolla, California, CA 92093 USA

**Keywords:** Malaria, Transmission, PCR, Heterogeneity, Hotspot, Peruvian Amazon

## Abstract

**Background:**

Understanding the dynamics of malaria transmission in diverse endemic settings is key for designing and implementing locally adapted and sustainable control and elimination strategies. A parasitological and epidemiological survey was conducted in September–October 2012, as a baseline underlying a 3-year population-based longitudinal cohort study. The aim was to characterize malaria transmission patterns in two contrasting ecological rural sites in the Peruvian Amazon, Lupuna (LUP), a riverine environment, and Cahuide (CAH), associated with road-linked deforestation.

**Methods:**

After a full population census, 1941 individuals 3 years and older (829 in LUP, 1112 in CAH) were interviewed, clinically examined and had a blood sample taken for the detection of malaria parasites by microscopy and PCR. Species-specific parasite prevalence was estimated overall and by site. Multivariate logistic regression models assessed risk factors for parasite infection by PCR, while SaTScan detected spatial clusters of PCR-positive individuals within each site. In addition, data from routine malaria surveillance in the period 2009–2012 were obtained.

**Results:**

Parasite prevalence by PCR was higher in CAH than in LUP for *Plasmodium vivax* (6.2% vs. 3.9%) and for *Plasmodium falciparum* (2.6% vs. 1.2%). Among PCR-confirmed infections, asymptomatic (Asy) parasite carriers were always more common than symptomatic (Sy) infections for *P. vivax* (Asy/Sy ratio: 2/1 in LUP and 3.7/1 in CAH) and for *P. falciparum* (Asy/Sy ratio: 1.3/1 in LUP and 4/1 in CAH). Sub-patent (Spat) infections also predominated over patent (Pat) infections for both species: *P. vivax* (Spat/Pat ratio: 2.8/1 in LUP and 3.7/1 in CAH) and *P. falciparum* malaria (Spat/Pat ratio: 1.9/1 in LUP and 26/0 in CAH). For CAH, age, gender and living in a household without electricity were significantly associated with *P. vivax* infection, while only age and living in a household with electricity was associated with *P. falciparum* infection. For LUP, only household overcrowding was associated with *P. falciparum* infection. The spatial analysis only identified well-defined clusters of *P. vivax* and *P. falciparum* infected individuals in CAH. Reported malaria incidence indicated that malaria transmission has long occurred in LUP with primarily seasonal patterns, and confirmed a malaria outbreak in CAH since May 2012.

**Conclusions:**

This parasitological and epidemiological baseline assessment demonstrates that malaria transmission and parasite prevalence is heterogeneous in the Peruvian Amazon, and influenced by local socio-demographics and ecological contexts. Riverine and road construction/deforestation contexts must be taken into account in order to carry out effective anti-malaria control and elimination efforts.

**Electronic supplementary material:**

The online version of this article (doi:10.1186/s12936-017-1957-y) contains supplementary material, which is available to authorized users.

## Background

Malaria remains an important public health problem in Peru, especially in the Amazonian department of Loreto where most malaria occurs [1–4]. Following the resurgence of malaria in the 1990s [5–8], which reached an historic peak of 158,115 cases in 1997 [9], the Loreto Regional Directorate of Health (LRDH) and national Peruvian Ministry of Health (MoH) organized control efforts that were intensified with the support of international agencies, particularly the Global Fund for AIDS, Tuberculosis and Malaria (GFATM) and the US Agency for International Development (USAID) [10]. Comprehensive community-based interventions were initiated and intensified that included: delivery of long-lasting insecticidal mosquito nets (LLINs) [11], environmental management with community participation, training of community health workers and microscopists [12], and “test and treat” interventions incorporating artemisinin-based combination therapy (ACT) for *Plasmodium falciparum*. It is thought that these interventions contributed to a reduction of microscopically-confirmed malaria cases in Loreto, from 54,291 cases in 2005 to 10,504 and 11,793 cases in 2010 and 2011, respectively [2, 13], and to an increasingly low-level, residual transmission in the region [2, 14].

Routine malaria control activities in the Peruvian Amazon are based on passive case detection (PCD) using light microscopy (LM) [2, 15] on patients presenting with fever or malaria-compatible illness at local health facilities. A positive LM result leads to government-provided, species-specific anti-malarial treatment. In areas with low and residual transmission, active, household- or field-based malaria surveillance is challenging because most infected individuals have very low-level parasitaemia, often exceeding the limit of detection of LM [16–18]. Indeed, several cross-sectional surveys in rural villages surrounding Iquitos city (capital of Loreto) have reproducibly demonstrated that the prevalence of asymptomatic parasite carriers ranges from 4 to 15%, and that molecular diagnostic tools such as polymerase chain reaction (PCR) is far more sensitive than LM [14, 19–21] for identifying the presence of parasitaemia. Villages in those studies were located along the road connecting Iquitos to the Nauta district, thus presenting varying degrees of development-driven deforestation associated with increased human-biting activity of *Anopheles darlingi* (the main malaria vector in the Peruvian Amazon) [8]. Collectively these observations suggest that asymptomatic carriers are a hidden human infectious reservoir that, combined with favourable ecological conditions for mosquito breeding and resting sites around places where people live or work, maintain local malaria transmission in Amazonia, hence hypoendemicity and residual malaria [22–25].

The hypothesis that asymptomatic *Plasmodium vivax* and *P. falciparum* parasitaemias contribute to the maintenance of malaria hypoendemicity in the Amazon region drives the work of our Amazonia International Center of Excellence in Malaria Research (Amazonia ICEMR), which was established in 2010 as one of ten such centers supported by the National Institute of Allergy and Infectious Diseases (NIAID) of the National Institutes of Health (NIH) [26]. The Amazonia ICEMR is conducting population-based longitudinal cohort studies with the aim of assessing how sub-microscopic, asymptomatic infections contribute to maintaining transmission in epidemiological contrasting settings of the Peruvian Amazon [27]. This paper focuses on the socio-demographics and epidemiological characteristics of two contrasting exemplars of complex malaria transmission in the Peruvian Amazon, the village of Cahuide, a rural site with road-associated deforestation, and Lupuna rural site with riverine environment. Population-based estimates of *P. vivax* and *P. falciparum* prevalence by LM and PCR at the baseline enrollment surveys in September–October 2012 are also discussed and those estimates were related to data from routine malaria surveillance and implemented control efforts to better understand the dynamics of malaria transmission in both sites.

## Methods

### Ethics statement

Ethics clearance for the study was obtained from the Ethics Review Board of the Universidad Peruana Cayetano Heredia, Lima, Peru (SIDISI code # 5739) and from the University of California San Diego Human Subjects Protection Program (Project # 100765). Permissions were received from health and local authorities after explaining the purpose and procedures of the study. Signed informed consent was obtained prior the study enrollment to participation and blood sampling by all adults and the parents of all participating children <18 years. In addition to parental/guardian consent, children older than 7 years provided a signed informed assent. All the methods were carried out in accordance with approved guidelines.

### Study design

A census and an epidemiological survey were conducted as baseline activities of a 3-year population-based, longitudinal cohort study in the Lupuna (LUP) and Cahuide (CAH) sites in the northeastern Peruvian Amazon Department of Loreto. The study population included individuals aged 3 years and older who resided in study sites and agreed to participate in the cohort providing a written signed consent.

### Study area

**Fig. 1:**
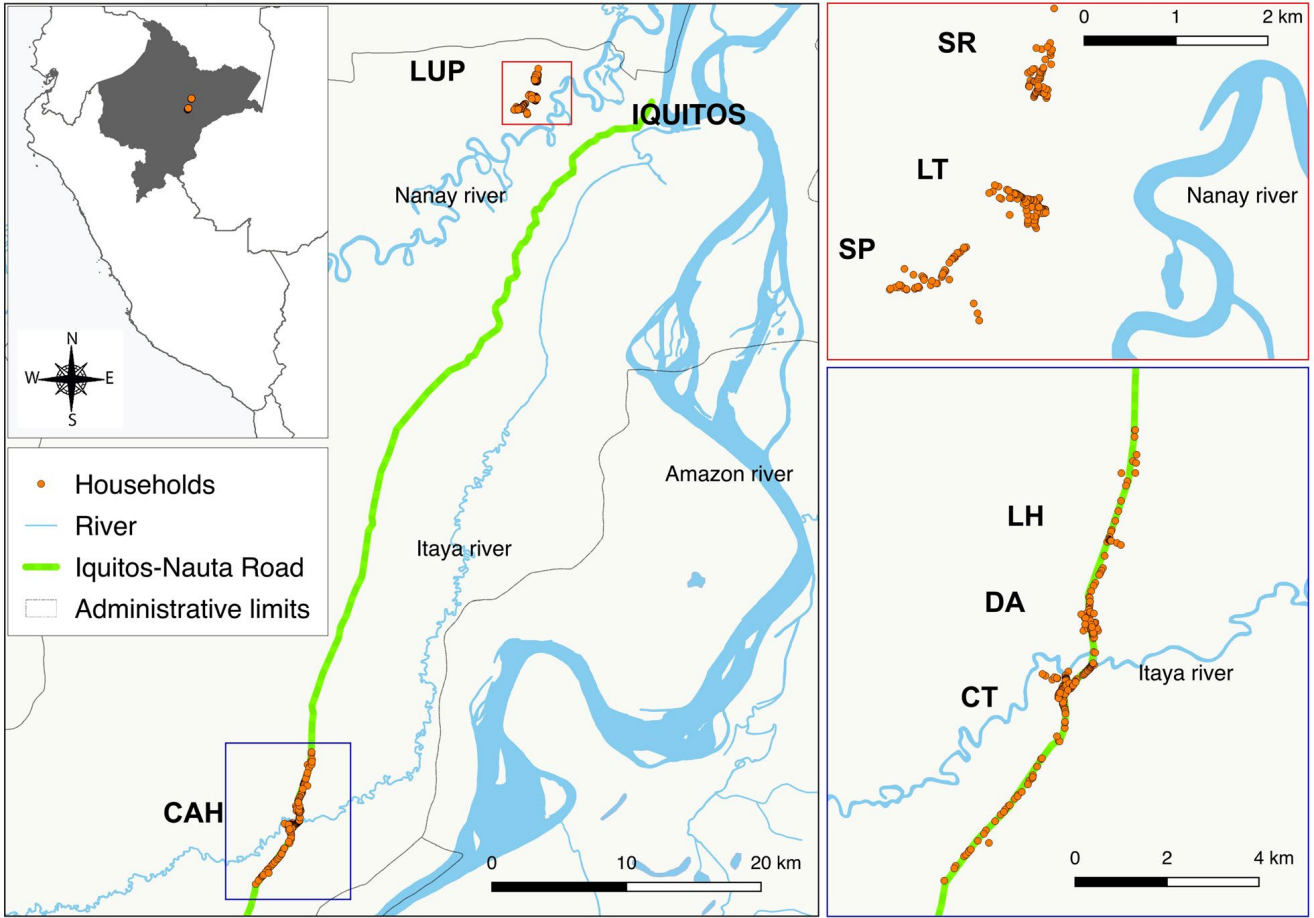
Study area. Sites: Lupuna (LUP), Cahuide (CAH). Villages: Santa Rita (SR), San Juan de Lupuna town (LT), San Pedro (SP), La Habana (LH), Doce de Abril (DA), Cahuide town (CT)

LUP site is located on the outskirts of Iquitos district, 10 km from downtown (latitude 03°44.591′S longitude 73°19.615′W) (Fig. 1). It is a forested area only accessible by crossing the Nanay River by boat. LUP is comprised of three contiguous villages, Santa Rita (SR), San Jose de Lupuna town (LT), and San Pedro (SP) situated about 500–1000 m from the riverbanks near the administrative border between San Juan and Iquitos districts. Villagers mainly work in agricultural activities, such as cassava cultivation and charcoal production. CAH includes three rural villages in the southern part of San Juan district, La Habana (LH), Doce de Abril (DA) and Cahuide town (CT), whose houses are located on both sites of the Iquitos-Nauta road between the 54th and 63th km. These three villages were established in the 1980s after the intensification of deforestation and extension of the road connecting Iquitos city to the Nauta district. CT, the most populated CAH village, is a centre of palm roof production, and has a port close to the intersection between the road and the Itaya River (around 58th km, latitude 04°13.785′S longitude 73°276′W). The climate of study area is tropical, warm and humid with a rainy season from November to May and a dry season from June to October [14]. Annual average temperature is around 27 °C, the relative humidity above 80%, with average annual rainfall of 4 m. *Anopheles darlingi* is the primary malaria vector in the area [28].

*Plasmodium vivax* and *P. falciparum* cases are reported to the Ministry of Health annually for both sites throughout the entire year [28]. As in most Peruvian endemic areas, malaria surveillance in study sites relies on passive case detection (PCD). Patients presenting with fever or illness compatible with malaria are systematically tested by LM at Ministry of Health-supported LUP and CAH health posts located in LT and CT, respectively. Microscopic diagnosis is available during weekdays between 8 a.m. and 2 p.m., but not during the weekend. *Plasmodium vivax* malaria was treated with chloroquine (CQ) for 3 days (10 mg/g on days 1 and 2, and 5 mg/kg on day 3), plus primaquine (PQ) for 7 days (0.5 mg/kg/day); *P. falciparum* malaria was treated with mefloquine (MQ) (12.5 mg/kg/day for 2 days) plus artesunate (AS) (4 mg/kg/day for 3 days), according to Peruvian national policy.

### Census and geo-referencing

A complete census of the population in each village was conducted in July–August 2012, collecting individual data on socio-demographics (e.g. age, gender, education, occupation, socio-economic status, time living in the village), and on previous malaria episodes. Each house was identified with a unique number and geo-referenced using a handheld Global Positioning System (GPS) device (Garmin’s GPSMAP 60CSx, Garmin International Inc., USA), and household characteristics collected (e.g. household size, predominant construction materials in house, ownership of animals, and availability of essential services such as potable water, sewage system and electricity). Each individual subject was given a seven-digit unique code number combining the village, household and individual code.

### Cohort enrollment and baseline survey

Individuals aged 3 years and older available at the time of the baseline survey (September–October 2012) were enrolled in the study area after consenting. Whenever selected individuals were absent, the household was revisited up to four times in a period of 30 days to maximize subject participation. Each participant had the axillary temperature taken, history of fever or any other malaria symptoms were recorded, and a finger-prick blood sample collected for immediate microscopy (thick and thin blood smears) and on filter paper (Whatman grade 3, Whatman, Springfield Mill, USA) for later molecular tests. Filter paper dried blood samples were individually stored at 4 °C with desiccant until processed. Subjects with microscopically-confirmed infections were referred to health posts for treatment according to Peruvian national guidelines.

### Retrospective routine surveillance data

Additionally, routine surveillance data, namely reported malaria episodes by species in Iquitos and San Juan district from 2009 to 2012 were obtained from the Regional Directorate of Health, Loreto-Ministry of Health in Iquitos. Peruvian policy mandates weekly notification of confirmed malaria cases (by standard LM) at health facilities and data aggregation at district and department level. Since malaria surveillance data could be reliably disaggregated only to the district level but not to the village level, a retrospective review of all registered individuals detected by PCD between January 2009 and December 2012 was carried out in all health facilities near the study sites. Any other surveillance in addition to routine PCD conducted in CAH and LUP in the 12 months before the survey was recorded.

### Laboratory procedures

#### Microscopy

Thick and thin smears were stained for 10 min with a 10% Giemsa solution, and parasite density was calculated by counting the number of asexual parasites for 200 white blood cells (WBC) in the thick smear, assuming a concentration of 8000 WBCs/μl. Slides were read on site, then a day later by an expert at microscopy at the reference laboratory in Iquitos. A slide was declared negative if no malaria parasite was found after examining 100 fields [29]. Quality control was done blindly on all positive slides and 10% of randomly chosen negative slides by a senior technician at Universidad Peruana Cayetano Heredia, Instituto de Medicina Tropical-“Alexander von Humboldt” in Lima, Peru.

#### Real time PCR

Filter paper blood spots were cut into ~6 mm^2^ pieces for DNA extraction using QIAamp DNA Microkit of QIAGEN following the manufacturer instructions. A quantitative real-time polymerase chain reaction (qPCR) method targeting the 18s rRNA gene region was used for molecular diagnosis, following the protocol reported by Mangold et al. [30] Primers were 5-TAACGAACGAGATCTTAA-3 and 5-GTTCCTCTAAGAAGCTTT-3. Cycling conditions were initial denaturation at 95 °C for 2 min, followed by amplification for 45 cycles of 20 s at 95 °C, 20 s at 52 °C, and 30 s at 68 °C. To confirm amplicon identify, amplification was immediately followed by a melt program consisting of 5 s at 65 °C and a stepwise temperature increase of 0.5 °C/s to 95 °C. Melting curves accurately differentiated *P. vivax* from *P. falciparum*.

### Data analysis

Census and survey data were doubly entered and cross-checked in Access (Microsoft Corp, USA). Data analysis was performed using R v.2.15 software (R Development Core Team, R Foundation for Statistical Computing, Austria). Baseline characteristics between sites were compared using the Chi squared test. Species-specific parasite prevalence by LM and by PCR were estimated overall and by site, and their two-sided 95% confidence intervals (CI) were calculated using the Wilson score method.

The case definition for malaria parasite (*Plasmodium* spp.) infection was an individual with a positive PCR result regardless of symptoms. Cases were further classified as patent (detectable by LM) or sub-patent (not detectable by LM). A symptomatic infection was defined as an individual with confirmation of parasites in the blood sample at any level who also had fever or history of fever, headache, chills or general discomfort in the previous 7 days. Uni- and multivariate mixed-effects logistic regression models, with fixed effects and a random intercept that accounted for individual clustering at household, were used to determine risk factors for species-specific malaria infection in each site The following potential risk factors were assessed: age, gender, time living in the village, education level, forest-related job as main economical activity (including the intensity of these activities during the week), trip in the past month to another endemic area, the history of malaria in the past year, household overcrowding (more than three individuals sleeping per room as average in a household), low household bed net coverage (<80% of beds covered with a net in a household), housing structure (predominant material in roof and floor), and the availability of electricity. Factors with *p* values <0.1 for the Wald test in the univariate analysis were considered for inclusion in the multivariate model. Using manual backward, final models retained all factors that were significantly associated with malaria infection (Wald *p* values <0.05). Interactions were systematically checked for up to order two. Likelihood ratio tests (LRTs) were used to assess statistical differences between nested models.

The QGIS software QGIS v.2.16 (QGIS developer team, Open Source Geospatial Foundation) was used to map all surveyed households and to classify them according to the number of household members with malaria infections identified by PCR. The SaTScan software v.9.3 (M Kulldorff and Information Management Services Inc, USA) was used to identify spatial clustering of households with malaria infections in each site, using the following characteristics: pure spatial analysis, Poisson probability model [31], latitude/longitude coordinates, report of most likely clusters with no geographical overlap of secondary clusters, maximum spatial cluster size equal to 50% of total population. The analysis was first done without adjustment for covariates, and then done including the variables identified as fixed-effect risk factors by the mixed-effects logistic regression analyses. To adjust for covariates, the number of malaria confirmed infections and the total number of screened individuals had to be specified for each particular household location and combination of covariates in the case and population files, respectively. SaTScan applied multiple circular windows across the study area, each circle representing a possible cluster. Clusters were assessed based on 999 Monte Carlo simulations to determine the probability of observed frequency of infected individuals being due to chance relative to expected frequency under the null hypothesis of no clustering. The null hypothesis was rejected if any resulting *p* value of assessed clusters was <0.05 and the window with the maximum log likelihood ratio (LLR) was identified as the most likely cluster. The relative risk (RR) reported for each identified cluster was the estimated risk within the cluster divided by the estimated risk outside the cluster [31].

## Results

A total of 487 households (HHs) and 2447 individuals were identified during the census in July–August 2012, distributed over the two sites as follows: LUP (211 HHs; 1007 individuals) and CAH (276; 1440). In addition, the distribution by village was: LT (95 HHs; 433 individuals), SR (66; 348) and SP (50; 226) for LUP, and CT (173; 922), LH (46; 224) and DA (57; 294) for CAH (Table 1). The overall ratio of female to male was 0.9; more than half of the population in both sites was younger than 25 years.

**Table 1:** Household and participants in census and baseline survey

	Census		Survey			
	HH	Individuals	HH		Individuals	
	N	N	n	%	n	%
Lupuna (LUP)						
Lupuna town (LT)	95	433	88	92.6	352	81.3
Santa Rita (SR)	66	348	63	95.5	292	83.9
San Pedro (SP)	50	226	45	90.0	185	81.9
Total	211	1007	196	92.9	829	82.3
Cahuide (CAH)						
Cahuide town (CT)	173	922	163	94.2	734	79.6
La Habana (LA)	46	224	37	80.4	147	65.6
Doce de Abril (DA)	57	294	52	91.2	231	78.6
Total	276	1440	252	91.3	1112	77.2

The cohort study enrolled 1941 of the 2447 censused inhabitants (79.3%) in 448 HH (92.0%) available at the time of the baseline survey. Of these 1941 individuals, 829 (42.7%) lived in LUP and 1117 (57.3%) in CAH. Socio-demographic characteristics and history of past malaria episodes by site and by village are presented in Table 2 and Additional file 1: Table S1, respectively. Individuals under 25 years represented 49.3 and 54.5% of the participants respectively in LUP and CAH (*p* = 0.008). Males slightly outnumbered females (ratio female/male = 0.89) without differences between sites. The proportion of participants aged >10 years old who have resided in the site ten or more years was significantly higher in LUP (85.0%) than in CAH (41.3%) (*p* < 0.001). Among adults 18 years and older, the proportion of individuals who had completed secondary education level was higher in LUP (47.0%) than in CAH (32.8%) (*p* < 0.001). The most commonly reported occupation among adults was farmers, followed by housewives, indoor laborers/employees and traders with similar proportions in both sites; security guards (*p* = 0.002) and loggers (*p* < 0.001) were more frequent in CAH than in LUP. Participants reporting having experienced malaria in their lifetime were more common in LUP (73.4%) than in CAH (67.0%) (p = 0.003). However, this was not the case when only recent episodes were considered, with more participants reporting have had malaria episodes within the past 12 months in CAH (48.9%) than in LUP (14.1%) (*p* < 0.001).

**Table 2:** Baseline socio-demographic characteristics of study participants

	Lupuna (LUP)		Cahuide (CAH)		Total	
	n	%	n	%	n	%
Gender						
Female	406	49.0	510	45.9	916	47.2
Male	423	51.0	602	54.1	1025	52.8
Age (years)*						
<5	55	6.6	88	7.9	143	7.4
5–14.9	196	23.6	336	30.3	532	27.5
15–24.9	158	19.1	191	17.2	349	18.0
25–39.9	159	19.2	215	19.4	374	19.3
40–54.9	132	15.9	164	14.8	296	15.3
>55	129	15.6	115	10.4	244	12.6
Missing	0		3		3	
Time in village (age ≥ 10 years)*						
<2	26	3.9	207	24.6	233	15.4
2–9.9	74	11.1	288	34.2	362	24.0
≥10	567	85.0	348	41.3	915	60.6
Missing	3		3		6	
Education (age ≥ 18 years)*						
None	21	4.0	25	4.1	46	4.0
Incomplete primary	130	24.9	200	32.6	330	29.0
Complete primary	126	24.1	187	30.5	313	27.6
Secondary	226	43.2	185	30.2	411	36.2
Superior	20	3.8	16	2.6	36	3.2
Missing	3		3		6	
Main occupation (age ≥ 18 years)*						
None	29	5.6	21	3.4	50	4.4
Student	24	4.6	9	1.5	33	2.9
Housewife	124	23.9	145	23.7	269	23.8
Trader	47	9.1	65	10.6	112	9.9
Labourer, employee	72	13.9	88	14.4	160	14.2
Guardian	2	0.4	43	7.0	45	4.0
Farmer	178	34.4	180	29.4	358	31.7
Logger	22	4.2	55	9.0	77	6.8
Fisher, hunter	13	2.5	5	0.8	18	1.6
Boat driver	7	1.4	1	0.2	8	0.7
Missing	8		4		12	
Lifetime malaria episodes*						
0	215	26.6	365	33.0	580	30.3
1	133	16.5	314	28.4	447	23.3
2–3	175	21.7	271	24.5	446	23.3
≥4	285	35.3	157	14.2	442	23.1
Missing	21		5		26	
Malaria episodes (previous 12 months)*						
0	704	85.9	566	51.1	1270	65.9
1	93	11.3	429	38.7	522	27.1
2–3	22	2.7	100	9.0	122	6.3
≥4	1	0.1	13	1.2	14	0.7
Missing	9					

* Significant difference between sites (p < 0.05)

Table 3 and Additional file 1: Table S2 present the household characteristics of enrolled participants by site and by village, respectively. Household crowding was higher in CAH (57.6%) than in LUP (41.9%) (*p* < 0.001). In both sites, most individuals lived in houses with wooden walls and roofs composed of palm leaf; concrete (brick and cement) and tin were also common building materials, especially in the LT village of LUP (*p* < 0.001) (Additional file 1: Table S2). Wooden floors built on stilts predominated in houses of CAH, while dirt floors were more frequent in LUP, especially in SR (*p* < 0.001). Most participants had electricity at home in CAH (55.7%), primarily those living along the road and in the DA village (*p* < 0.001); however this electrical service mainly exclusively benefited participants living in LT village in LUP (*p* < 0.001). The use of potable water for drinking and food preparation was reported by only a small proportion of individuals in LUP (15.6%) and CAH (30.7%), while firewood was reported as the main fuel used for cooking by most participants (>80%) in both sites. Moreover, most participants in both sites reported having precarious or lack of sanitation facilities, as well as lack of trash disposal systems. The majority (97.0%) of participants in CAH reported that their houses were sprayed indoors with insecticide in the past 3 months, while only 12.4% reported this in LUP (*p* < 0.001). Household bed net coverage was high in both sites; the predominant material of bed nets reported by participants varied across sites. In all villages of LUP, most bed nets (about 90%) were LLINs. In CAH, about half of subjects reported having LLINs and half reported using non-impregnated insecticide nets made of tocuyo (non-processed cotton) or nylon.

**Table 3:** Baseline household characteristics of study participants

	Lupuna (LUP)		Cahuide (CAH)		Total	
	n	%	n	%	n	%
Overcrowding (>3 persons/bedroom)*						
No	482	58.1	471	42.4	953	49.1
Yes	347	41.9	639	57.6	986	50.9
Missing	0		2		2	
Wall material*						
Brick, cement	130	15.7	51	4.6	181	9.3
Wood	629	75.9	919	82.8	1548	79.8
Palm	47	5.7	61	5.5	108	5.6
Tin, other	23	2.8	79	7.1	102	5.3
Missing	0		2		2	
Roof material*						
Tin	271	32.7	157	14.2	428	22.1
Palm	558	67.3	947	85.8	1505	77.9
Missing	0		8		8	
Floor material*						
Cement	203	24.5	141	12.7	344	17.7
Wood	98	11.8	640	57.7	738	38.1
Dirt	528	63.7	329	29.6	857	44.2
Missing	0		2		2	
Electricity*						
Yes	298	35.9	618	55.7	916	47.2
No	531	64.1	492	44.3	1023	52.8
Missing	0		2		2	
Potable water for drinking*						
Yes	129	15.6	337	30.7	466	24.2
No	700	84.4	760	69.3	1460	75.8
Missing	0		15		15	
Source of water*						
Piped into dwelling	6	0.7	44	4.1	50	2.6
Public tap	272	32.8	292	27.5	564	29.8
Open well	309	37.3	358	33.7	667	35.3
River, rain	242	29.2	368	34.7	610	32.3
Missing	0		50		50	
Sanitation facility*						
Flush toilet	4	0.5	3	0.3	7	0.4
Pit latrine	168	20.4	306	27.6	474	24.5
Ground hole, cesspool	279	33.9	518	46.7	797	41.2
No facility, field	373	45.3	283	25.5	656	33.9
Missing	5		2		7	
Trash disposal*						
Burning trash	240	29.0	467	42.4	707	36.6
Bury trash	56	6.8	163	14.8	219	11.3
Field, river	510	61.5	466	42.3	976	50.6
Other	23	2.8	5	0.5	28	1.5
Missing	0		11		11	
Cooking fuel*						
Gas	22	2.7	40	3.6	62	3.2
Kerosene, charcoal	13	1.6	137	12.4	150	7.7
Firewood	793	95.8	932	84.0	1725	89.1
Missing	1		3		4	
Insecticide sprayed (previous 3 months)*						
Yes	93	12.4	988	97.0	1081	61.1
No	656	87.6	31	3.0	687	38.9
Missing	80		93		173	
Bednet coverage (bednets/beds), %						
<80	28	3.4	32	2.9	60	3.1
≥80	801	96.6	1078	97.1	1879	96.9
Missing	0		2		2	
Bednet material*						
None	0	0.0	2	0.2	2	0.1
Tocuyo	41	4.9	400	36.1	441	22.8
Nylon	33	4.0	124	11.2	157	8.1
LLINs	755	91.1	557	50.2	1312	67.7
Other	0	0.0	26	2.3	26	1.3
Missing	0		3		3	

* Significant difference between sites (p < 0.05)

All 1941 individuals enrolled in the cohort had samples obtained for malaria diagnosis by LM at the time of the baseline survey; most of these (1770 individuals, 92.2%) had available sample for PCR analysis. By LM, the overall parasite prevalence was 2.1% (38 *P. vivax*, 2 *P. falciparum* infections), and by PCR 7.2% (93 *P. vivax*, 35 *P. falciparum*) (Table 4), with the highest figures in CAH site *(p* < 0.05). Of the total 38 *P. vivax* infections by LM, 30 were found in CAH (16 with parasitaemia <100 parasites/μl), and only 8 in LUP (one with parasitaemia <100 parasites/μl); while the two microscopically confirmed *P. falciparum* infections were found in LUP (one with parasitaemia <100 parasites/μl). The presence of gametocytes was recorded in 15 (50.0%) and 3 (37.5%) of the total microscopically confirmed *P. vivax* infections in CAH and LUP, respectively. Gametocytes were not observed in microscopically confirmed *P. falciparum* infections. Parasite prevalence by PCR was higher in CAH than in LUP for *P. vivax* (6.2% vs. 3.9%, p = 0.03) and for *P. falciparum* (2.6% vs. 1.2%, p = 0.03). Considering PCR-confirmed infections, asymptomatic (Asy) parasite carriers were always more common than symptomatic (Sy) infections for *P. vivax* (Asy/Sy ratio: 2/1 in LUP and 3.7/1 in CAH) and for *P. falciparum* (Asy/Sy ratio: 1.3/1 in LUP and 4/1 in CAH). Similarly, sub-patent (Spat) infections also predominated over patent (Pat) infections for both species: *P. vivax* (Spat/Pat ratio: 2.8/1 in LUP and 3.7/1 in CAH) and *P. falciparum* malaria (Spat/Pat ratio: 1.9/1 in LUP and 26/0 in CAH).

**Table 4:** Malaria prevalence (by LM and PCR) by study site

	Lupuna (LUP)				Cahuide (CAH)				Total			
	n	%	% [95% CI]		n	%	% [95% CI]		n	%	% [95% CI]	
Microscopy												
Total analysed samples (N)	829				1112				1941			
P. vivax*	8	1.0	0.4	2.0	30	2.7	1.9	3.9	38	2.0	1.4	2.7
*P. falciparum*	2	0.2	0.0	1.0	0	0.0			2	0.1	0.0	0.4
Overall*	10	1.2	0.6	2.3	30	2.7	1.9	3.9	40	2.1	1.5	2.8
Asymptomatic *P. vivax*	3	0.4	0.1	1.1	24	2.2	1.4	3.2	27	1.4	0.9	2.0
Symptomatic *P. vivax*	5	0.6	0.2	1.5	6	0.5	0.2	1.2	11	0.6	0.3	1.0
Asymptomatic *P. falciparum*	1	0.1	0.0	0.8	0	0.0			1	0.1	0.0	0.3
Symptomatic *P. falciparum*	1	0.1	0.0	0.8	0	0.0			1	0.1	0.0	0.3
PCR												
Total analysed samples (N)	777				1013				1790			
P. vivax*	30	3.9	2.7	5.5	63	6.2	4.8	7.9	93	5.2	4.2	6.4
P. falciparum*	9	1.2	0.6	2.3	26	2.6	1.7	3.8	35	2.0	1.4	2.7
Overall*	39	5.0	3.6	6.9	89	8.8	7.2	10.7	128	7.2	6.0	8.5
Asymptomatic *P. vivax*	20	2.6	1.6	4.0	53	5.2	4.0	6.8	73	4.1	3.2	5.1
Symptomatic *P. vivax*	10	1.3	0.7	2.4	10	1.0	0.5	1.9	20	1.1	0.7	1.8
Asymptomatic *P. falciparum*	5	0.6	0.2	1.6	23	2.3	1.5	3.4	28	1.6	1.1	2.3
Symptomatic *P. falciparum*	4	0.5	0.2	1.4	3	0.3	0.1	0.9	7	0.4	0.2	0.8
Sub-patent *P. vivax*	22	2.8	1.8	4.3	41	4.0	3.0	5.5	63	3.5	2.7	4.5
Patent *P. vivax*	8	1.0	0.5	2.1	22	2.2	1.4	3.3	30	1.7	1.2	2.4
Sub-patent *P. falciparum*	8	1.0	0.5	2.1	26	2.6	1.7	3.8	34	1.9	1.3	2.7
Patent *P. falciparum*	1	0.1	0.0	0.8	0	0.0			1	0.1	0.0	0.4

* Significant difference between sites (p < 0.05)

Given the relatively small number of infections detected by LM, uni- and multivariate mixed-effects logistic regression analyses were carried out only for confirmed infections by PCR for each site. In final models the estimates of the random-effects coefficients confirmed the influence of clustered sampled data at household level. In LUP, household overcrowding (i.e., more than three individuals sleeping per room as average in a household) was the only factor significantly associated with *P. falciparum* infection (OR 4.9, 95% CI [1.0–24.6]). No significant factors were found for *P. vivax* infection (Table 5). In CAH, age, gender and electricity availability (a proxy for income) remained independently associated with *P. vivax* malaria infection in the multivariate model (Table 6). While individuals between 15 and 39 years (AOR 2.8, 95% CI [1.4–5.6]) and those older than 40 years (AOR 2.4, 95% CI [1.2–5.1]) had higher odds of *P. vivax* infection than those <15 years, male participants were two times more likely to have a *P. vivax* infection than female participants (AOR 2.0, 95% CI [1.1–3.6]). Moreover, the lack of electricity increased the odds of infection in household members by a factor of 1.9 (AOR 1.9, 95% CI [1.1–3.4]) compared with individuals living in households that had available this public service. Considering *P. falciparum*, the odds of infection confirmed by PCR significantly increased at younger age (test for trend *p* < 0.001), with individuals <15 years being four times more likely to have *P. falciparum* infections than adults >40 years (AOR 5.5, 95% CI [1.3–24.4]). In this case, the availability of electricity was associated with highest risk of *P. falciparum* in household members (AOR 2.8, 95% CI [1.1–7.4]) compared with individuals living in households without electricity. No association was found between *P. vivax* and *P. falciparum* infection with regard to time of residence in the village, education level, occupation, trip in the past month to another endemic area, the history of malaria in the past year, and housing characteristics of study participants.

**Table 5:** Univariate risk factor analysis for *P. vivax* and *P. falciparum* infection in Lupuna (LUP)

	*P. vivax* by PCR						*P. falciparum* by PCR					
	n	N	%	OR	OR [95% CI]		n	N	%	OR	OR [95% CI]	
Gender												
Female	13	382	3.4	Ref			3	382	0.8	Ref		
Male	17	395	4.3	1.3	0.6	2.7	6	395	1.5	1.9	0.5	7.7
Age (years)												
<15	9	234	3.8	Ref			5	234	2.1	6.3	0.7	58.7
15–39	15	291	5.2	1.4	0.6	3.2	3	291	1.0	2.7	0.3	27.6
≥40	6	252	2.4	0.6	0.2	1.7	1	252	0.4	Ref		
Time in village (years)												
<10	7	242	2.9	Ref			3	242	1.2	Ref		
≥10	23	535	4.3	1.5	0.6	3.6	6	535	1.1	0.9	0.2	3.7
Education												
None	3	89	3.4	Ref			1	89	1.1	Ref		
Incomplete primary	6	245	2.4	0.7	0.2	2.9	2	245	0.8	0.7	0.1	8.6
Complete primary	6	153	3.9	1.2	0.3	4.8	2	153	1.3	1.1	0.1	13.3
Secondary	15	290	5.2	1.6	0.4	5.5	4	290	1.4	1.2	0.1	11.8
Forest-related job												
No	21	563	3.7	Ref			7	563	1.2	Ref		
Yes (≤5 day/week)	8	153	5.2	1.4	0.6	3.3	2	153	1.3	0.9	0.2	4.9
Yes (>5 day/week)	0	56	0.0	NC			0	56	0.0	NC		
Trip previous month												
No	30	771	3.9	Ref			9	771	1.2	Ref		
Yes	0	6	0.0	NC			0	6	0.0	NC		
Malaria episodes (previous 12 months)												
0	25	662	3.8	Ref			9	662	1.4	Ref		
≥1 episodes	5	107	4.7	1.2	0.5	3.3	0	107	0.0	NC		
Overcrowding (>3 persons/bedroom)												
No	17	452	3.8	Ref			2	452	0.4	Ref		
Yes	13	325	4.0	1.1	0.5	2.2	7	325	2.2	4.9*	1.0	24.6
Bednet coverage, %												
≥80	30	751	4.0	Ref			9	751	1.2	Ref		
<80	0	26	0.0	NC			0	26	0.0	NC		
Roof material												
Calamine	9	245	3.7	Ref			2	245	0.8	Ref		
Palm	21	532	3.9	1.1	0.5	2.4	7	532	1.3	1.6	0.3	8.3
Floor material												
Cement	5	184	2.7	Ref			1	184	0.5	Ref		
Wood	6	93	6.5	2.5	0.7	8.3	0	93	0.0	NC		
Dirt	19	500	3.8	1.4	0.5	3.8	8	500	1.6	2.9	0.2	38.1
Electricity												
Yes	13	271	4.8	Ref			1	271	0.4	Ref		
No	17	506	3.4	0.7	0.3	1.4	8	506	1.6	4.3	0.5	36.0

Random-effects variance for *P. falciparum* model = 0.62 (including only overcrowding)

*Ref* reference, *NC* non calculated

* p < 0.05

**Table 6:** Univariate and multivariate risk factor analysis for *P. vivax* and *P. falciparum* infection in Cahuide (CAH)

	*P. vivax* by PCR									*P. falciparum* by PCR								
	n	N	%	OR	OR [95% CI]		AOR	AOR [95% CI]		n	N	%	OR	OR [95% CI]		AOR	AOR [95% CI]	
Gender																		
Female	19	471	4.0	Ref			Ref			12	471	2.5	Ref					
Male	44	542	8.1	2.1	1.2	3.8	2.0*	1.1	3.6	14	542	2.6	1.0	0.5	2.3			
Age (years)																		
<15	13	401	3.2	Ref			Ref			17	401	4.2	5.6*	1.3	24.8	5.5*	1.3	24.4
15–39	30	362	8.3	2.8*	1.4	5.5	2.8*	1.4	5.6	7	362	1.9	2.4	0.5	12.0	2.3	0.5	11.3
≥40	20	249	8.0	2.6*	1.2	5.5	2.4*	1.2	5.1	2	249	0.8	Ref			Ref		
Time in village (years)																		
<2	17	237	7.2	Ref						5	237	2.1	Ref					
2–9	26	448	5.8	0.8	0.4	1.7				15	448	3.3	1.6	0.5	4.6			
≥10	20	328	6.1	0.9	0.4	1.9				6	328	1.8	0.8	0.2	2.8			
Education																		
None	7	135	5.2	Ref						5	135	3.7	Ref					
Incomplete primary	23	397	5.8	1.2	0.5	3.0				10	397	2.5	0.7	0.2	2.0			
Complete primary	17	242	7.0	1.6	0.6	4.1				5	242	2.1	0.5	0.1	1.9			
Secondary	16	237	6.8	1.5	0.6	3.9				6	237	2.5	0.7	0.2	2.2			
Forest-related job																		
No	37	750	4.9	Ref			Ref			25	750	3.3	Ref					
Yes (≤5 day/week)	4	38	10.5	1.6	0.8	3.1	1.1	0.3	3.3	0	183	0.0	NC					
Yes (>5 day/week)	16	168	9.5	3.4*	1.6	7.2	1.1	0.6	2.3	1	79	1.3	0.4	0.1	3.4			
Trip previous month																		
No	61	969	6.3	Ref						26	969	2.7	Ref					
Yes	2	44	4.5	0.7	0.2	3.2				0	44	0.0	NC					
Malaria episodes (previous 12 months)																		
0	31	499	6.2	Ref						16	499	3.2	Ref					
≥1 episodes	32	512	6.3	1.0	0.6	1.7				10	512	2.0	0.6	0.3	1.3			
Overcrowding (>3 persons/bedroom)																		
No	23	425	5.4	Ref						14	425	3.3	Ref					
Yes	40	588	6.8	1.3	0.7	2.3				12	588	2.0	0.6	0.3	1.4			
Bednet coverage, %																		
≥80	60	985	6.1	Ref						26	985	2.6	Ref					
<80	3	28	10.7	2.1	0.5	8.8				0	28	0.0	NC					
Roof material																		
Calamine	14	144	9.7	Ref						7	144	4.9	Ref					
Palm	49	865	5.7	0.6	0.3	1.1				19	865	2.2	0.4	0.2	1.1			
Floor material																		
Cement	9	122	7.4	Ref						6	122	4.9	Ref					
Wood	32	590	5.4	0.7	0.3	1.6				14	590	2.4	0.5	0.2	1.3			
Dirt	22	301	7.3	1.0	0.4	2.5				6	301	2.0	0.4	0.1	1.3			
Electricity																		
Yes	26	560	4.6	Ref			Ref			20	560	3.6	2.7	1.1	7.1	2.8	1.1	7.4
No	37	453	8.2	1.9*	1.0	3.3	1.9	1.1	3.4	6	453	1.3	Ref			Ref		

Random-effects variance for adjusted *P. vivax* model = 0.48

Random-effects variance for adjusted *P. falciparum* model = 0.36

*Ref* reference, *NC* non calculated

* p < 0.05

**Fig. 2:**
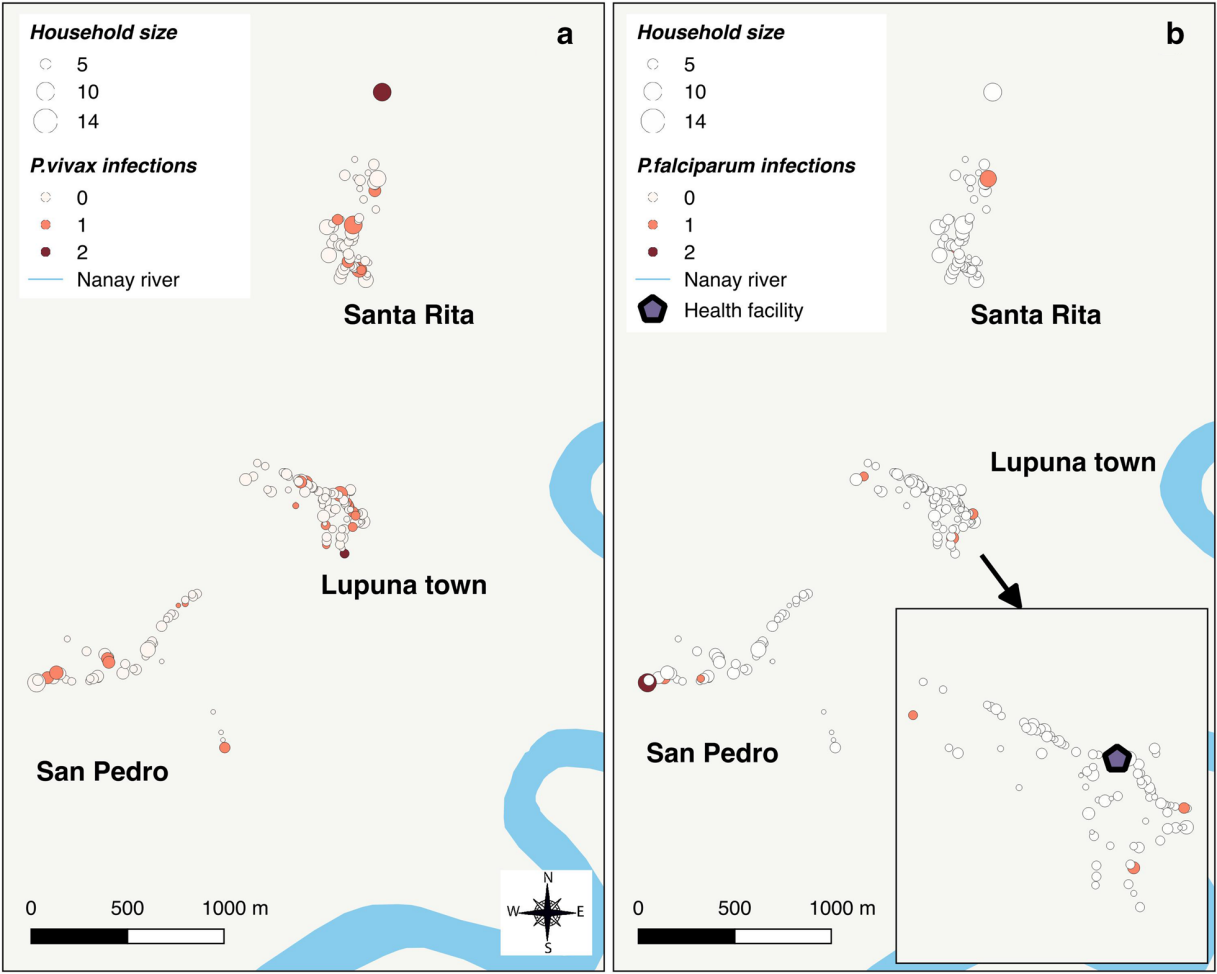
Distribution of *P. vivax-* (**a**) and *P. falciparum-* (**b**) infected individuals in Lupuna

**Fig. 3:**
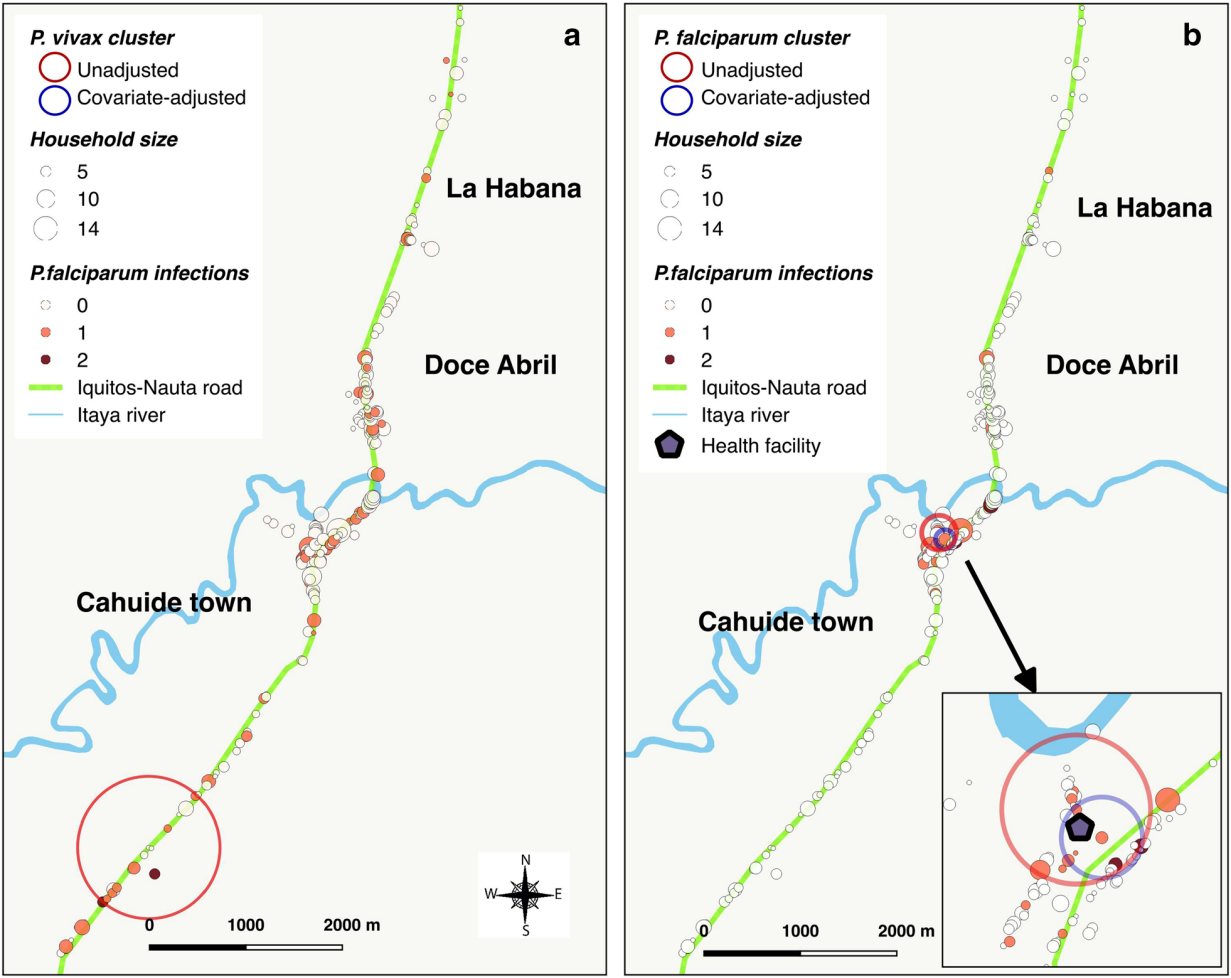
Distribution of *P. vivax*- (**a**) and *P. falciparum*- (**b**) infected individuals in Cahuide (CAH) with location of significant spatial clusters

Figures 2 and 3 show the spatial distribution of positive PCR individuals by species and for each site. Despite some aggregation of cases within adjacent houses in LUP, no significant spatial clustering of *P. vivax* and *P. falciparum* infections were identified using SaTScan (Fig. 2), mainly because of the low number of infections. However, spatial analysis demonstrated that species-specific malaria infection was non-randomly distributed in CAH. Indeed, the most likely spatial cluster of *P. vivax* infections (RR = 4.2, p = 0.028) was in the southern part of CT village (Fig. 3a), including 59 surveyed individuals with available PCR results (5.8% of total in CAH) in 16 households (6.6% of total). The cluster represented 20.6% (13/63) of all *P. vivax* confirmed infections by PCR. However, this cluster disappeared when the spatial analysis was adjusted for gender, age and electricity availability (factors associated with the infection according to risk factor analysis). For *P. falciparum*, the most likely cluster adjusted for age and electricity availability was the closest area to the fluvial port in CT (RR = 6.6, p = 0.04). With a radius of 99 meters, the cluster included 61 individuals (6.0% of the total) in 14 households (5.7% of the total) (Fig. 3b). This cluster represented 30.8% (8/26) of all *P. falciparum* confirmed infections by PCR. Notably, most individuals and households of the adjusted cluster were included within a previously identified bigger cluster (radius = 180 m) without adjustment for covariates.

**Fig. 4:**
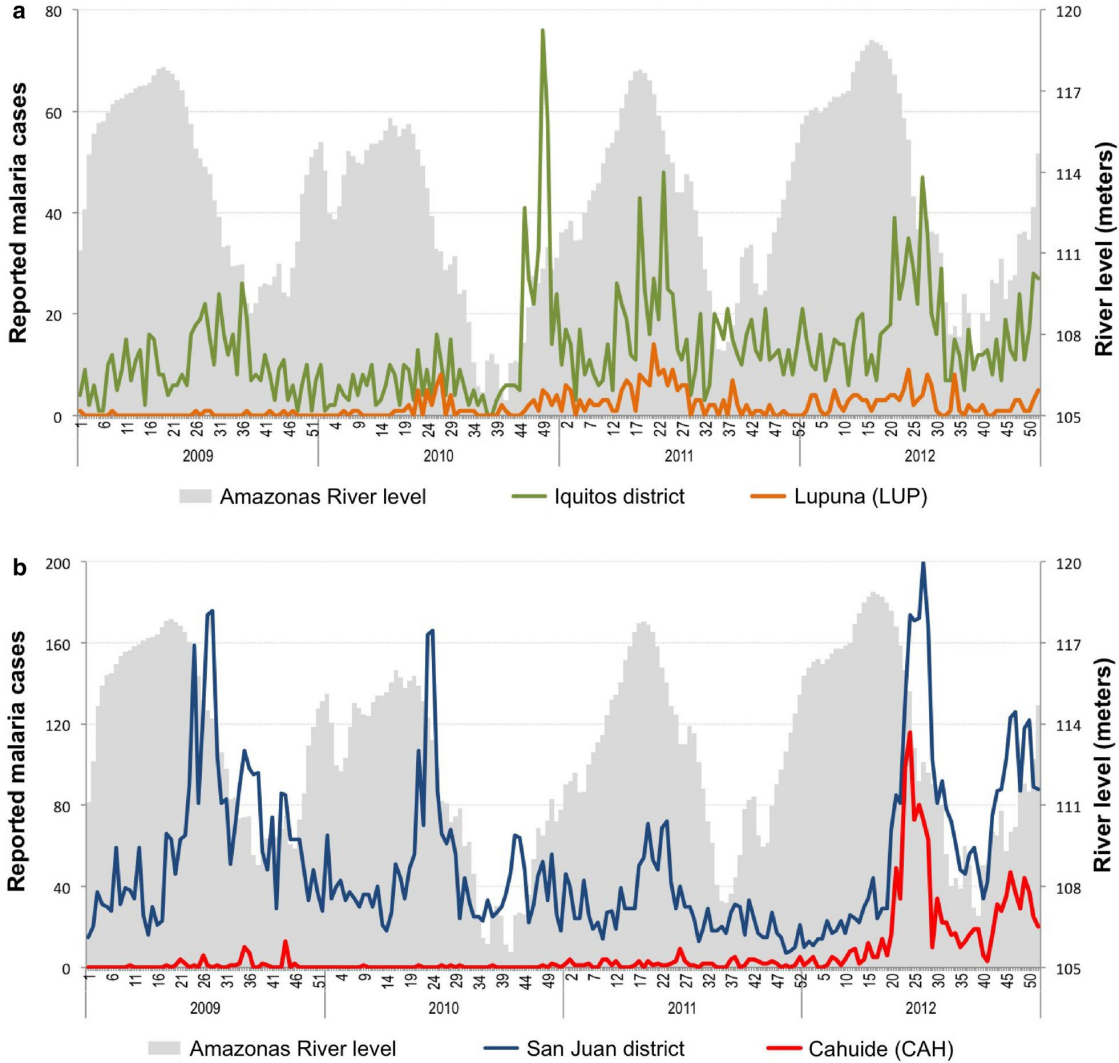
Weekly reported malaria cases in Lupuna (**a**) and Cahuide (**b**), and in their respective districts, Iquitos and San Juan (2009–2012)

The retrospective analysis of reported malaria cases between 2009 and 2012 showed that the malaria incidence in LUP varied across years, with some periods (mainly in year 2009) not following the same trends of malaria incidence compared to the Iquitos district (Fig. 4a). Despite this temporal heterogeneity in the malaria incidence in LUP, a seasonal increase of malaria cases was annually identified between April and July from 2010 and 2012. In 2012, malaria cases from LUP villages (124 *P. vivax*, 7 *P. falciparum*) represented about 15% of the total 880 reported cases in Iquitos district. Regarding malaria intervention efforts, deliveries of LLINs by GFATM’s PAMAFRO malaria project in 2008 and 2010 were the main malaria control intervention in LUP during the evaluated period. On the other hand, malaria cases in CAH between 2009 and 2011 occurred with stochastic patterns (with no more than 70 *P. vivax* and 10 *P. falciparum* reported cases per year), and did not follow the same trends of the malaria incidence than San Juan district during that period (Fig. 4b). The rapid increase of malaria cases in study villages of CAH since May 2012 following intensive and prolonged rains triggered comprehensive control efforts leaded by RHDL-MoH, such as successive mass-screening interventions with LM and treatment of confirmed infections (four rounds between May and August 2012), indoor spraying with residual insecticide (more than 90% of household coverage according to RHDL-MoH in May 2012), and the distribution of LLINs (one per household in July 2012) (Additional file 2: Figure S1). Households in CAH had previously received LLINs from PAMAFRO project only in 2007 [11], reaching high coverage levels. Although the outbreak was partially controlled by August 2012, malaria cases increased again in November–December 2012. At the end of the year, malaria cases (1221 *P. vivax* cases and 28 *P. falciparum* cases) from CAH villages accounted for about 35% of the total 3617 reported cases in San Juan district in 2012.

## Discussion

The baseline analysis presented here of a population-based, longitudinal malaria cohort study in two contrasting ecological villages in the Peruvian Amazon indicates substantial prevalence of malaria parasitaemia in the region and is an important benchmark for ongoing longitudinal studies. The riverine rural site, Lupuna (LUP), is home to a fairly stable population (85% of individuals >10 years having lived in the village 10 years or more). Here, the combined analysis of survey findings and routine reported malaria incidence suggests that malaria transmission has long occurred with primarily seasonal patterns. In contrast, Cahuide (CAH), a rural site with road-driven deforestation and a more transient population (about 60%), has experienced a notable and concerning increase of cases since May 2012 that compelled the regional government to carry out intensive outbreak control measures until August 2012 (1 month before the survey). Low parasite rates were observed in both sites during the survey, with high predominance of asymptomatic and sub-patent infections over symptomatic and patent infections. Moreover, the site-specific analysis of malaria prevalence and reported incidence suggested a high degree of heterogeneity at the micro-geographic level and over time for both *P. falciparum* and *P. vivax*.

Community surveys for determining malaria parasite carriage are commonly used to estimate malaria transmission in endemic areas [32, 33]. Because cross-sectional designs only include collecting data at one time point related to a specific outcome of interest (malaria), they are unable to capture the variations in malaria transmission over time (i.e., seasonal, epidemic or sporadic patterns in malaria transmission) [32]. Therefore, a better interpretation of the obtained prevalence rates in LUP and CAH requires understanding the appropriate context (riverine, road-associated) in Amazonia, and analysis of past trends in malaria incidence in relation with important events and factors influencing malaria transmission. Such analysis will be deepened by the longitudinally acquired data over the course of our ICEMR project (e.g., epidemiological, entomological, and ecological data), and will underlie the implementation of future control and elimination strategies.

The richness of these datasets is important to consider with regard to how future analyses will guide anti-malaria interventions. In riverine, rural LUP, malaria prevalence by PCR may indicate the seasonal-time dependent parasite carriage level in an endemic area (i.e., low seasonal transmission during the baseline survey) with no major recent changes in the population socio-demographics and in local control measures. In contrast, in road/deforestation and riverine rural CAH, prevalence of parasite carriers (with confirmed gametocytaemia by LM in some of them) reflects the parasite reservoir that enables persistence of malaria transmission by both *P. vivax* and *P. falciparum.* This was observed here to have occurred despite the intensive response from RHDL-MoH to a severe malaria outbreak from May to August 2012. These interpretations are further supported by a recently published entomological study of our Amazonian ICEMR that found that the highest monthly entomological inoculation rates (EIRs) are associated with seasonal transmission in both sites, with an important factor being seasonally high river levels that enable mosquito breeding [28]. Indeed, the entomological evaluation confirmed the presence of infected *An. darlingi* (by both *P. vivax* and *P. falciparum*) and higher human biting rates (HBRs) in LUP between February and June (maximum EIR in April) in successive years 2011 and 2012; while for CAH, the highest monthly EIR (2.52) with also confirmed *P. vivax*- and *P. falciparum*-infected *An. darlingi* occurred during the 2012 malaria outbreak. Interestingly, despite decreased HBR levels leading to a much lower estimated EIR in CAH in October 2012 (period of baseline survey), the entomological study found a high infection rate of collected mosquitoes (1.47%) which indicated onward malaria transmission in the area [28], and the potential new increase of cases if ecological conditions were favourable. This is exactly what happened in November–December 2012 [13] following the early onset of heavy rains generating an abrupt height increase of level rivers since October 2012, and consequently damaging and flooding to villages located along the Amazonas River and its tributaries [9].

As previously described elsewhere in the Peruvian Amazon [16–21], the findings presented here from both LUP and CAH consistently demonstrated that a large majority of malaria infections—by both *P. vivax* and *P. falciparum*—were asymptomatic (64–85%) and sub-microscopic (75–77%), despite differences in malaria prevalence and variations in malaria transmission over time. A limitation of such cross-sectional surveys is that they might not correctly estimate the proportion of infections that later might become symptomatic or have higher parasite density levels (as detectable by LM) [24, 34]. Few longitudinal studies in Amazonia, in particular Peru and Brazil, have provided data regarding such evolution [20, 22]. A 6-month cohort study in peri-Iquitos villages combining PCD, ACD and weekly monitoring of symptoms found that an important proportion of malaria infections which were asymptomatic at the time of detection (either by LM or PCR), developed malaria symptoms (reported and/or measured fever) after 1 week of follow-up, resulting in only 35–40% of asymptomatic infections among all confirmed cases in study villages [20, 34]. Another study conducted in western Brazilian Amazonia in 2004–2005 [22], including cross-sectional surveys, PCD and ACD over a 14-month period, found that among 93 asymptomatic infections confirmed by PCR during surveys, only 10 (10.7%) developed symptoms consistent with malarial disease over the subsequent 2 months of follow-up. Large prospective population cohorts with long and rigorous follow-up, as those recently implemented by the Amazonian ICEMR [26, 27], offer the opportunity to better delineate clinical and parasitological evolution of extant and/or newly acquired infections that were only detected by PCR. Population-based longitudinal study design is required to measure accurately the natural history of true and persistent asymptomatic and sub-microscopic carriers, and assess their contribution in maintaining malaria transmission in the Amazon region.

Considering the site-specific socio-demographics, household characteristics and ecological conditions in LUP and CAH, risk factor and spatial analyses of malaria infections were performed separately for each site. The low number of infections in LUP detected during the survey in low transmission season would primarily explain the difficulty in identifying malaria risk factors and spatial clusters of high transmission in the site for both *P. vivax* and *P. falciparum*. Household overcrowding, the only factor found to be significantly associated with *P. falciparum* infection in LUP, has also been reported in several studies conducted in areas where transmission is exclusively due to *P. falciparum*, or co-endemic with *P. vivax* [35, 36]. The effect of household overcrowding on malaria risk may be explained by the increase in human-released chemo-attractants for mosquitoes in reduced spaces [37], leading to higher human–vector contact rates. Despite the lack of association between *P. falciparum* infection and other proxy variables for socio-economic status in LUP, it is important to note that overcrowding can also reflect poor economic household conditions. The complex association between poverty and malaria is well known, which likely operates in both directions [38, 39]: poor households are less able to afford prevention and control measures and the higher burden of malaria may push these same individuals deeper into poverty.

Regardless of how the relationship of species-specific malaria with potential risk factors in CAH might be explained, it must be considered that our parasitological baseline survey probably captured the incipient onset of a second local outbreak of *P. vivax* and *P. falciparum* malaria. This outbreak likely included both: parasite carriers that remained despite the response to a first severe outbreak occurred between May and August 2012, and new infections by either similar or different parasite populations than those of the first outbreak. The increased risk for *P. vivax* in males and individuals aged >15 years together with the confirmed infection of mosquitoes in the site [28] suggests that transmission during the second outbreak may have been primarily determined by new infections (mostly asymptomatic infections with low parasitaemia) rather than by hypnozoite-triggered relapses. This explanation is further supported by entomological findings in the same site indicating that *An. darlingi* mosquitoes bite mainly outdoors [28], and by other studies in the Peruvian Amazon suggesting that the higher malaria risk of adult males is associated with increased human–vector contact rates following outdoor- and forest-related occupational activities (even though the latter activities were not resulted directly associated with the infection in the present study) [14, 40].

The differences in malaria risk between species with respect to the availability of electricity at household can be explained by the different connotations that this public service can have. While the lack of electricity may reflect poor socio-economic status associated with increased *P. vivax* infections and consequently suggest that transmission is not entirely outdoors; the electricity availability as risk factor for *P. falciparum* may be related with an increase of evening outdoor activities (e.g., watching television, chatting, resting, etc.) of household members [41]. On the other hand, the development of host immunity with the intensity of exposure to *P. falciparum* species in CAH (site that reports *P. falciparum* cases every year) may explain the significant relationship of *P. falciparum* infections with younger age groups [42–44], as well as, the finding that all *P. falciparum* infections had low parasite densities non-detectable by LM [16]. The results presented here from CAH differ from previous reports showing that prevalence and/or incidence of *P. vivax* decreased significantly faster with age than that of *P. falciparum*, suggesting that acquiring clinical immunity is different species-specific, as has been seen in other co-endemic countries such as Papua New Guinea [45], Thailand [46], Vanuatu [47], Ethiopia [48], and Brazil [42, 49]. The data presented here regarding LM−, PCR+ malaria infection do need to be interpreted with caution, considering the limitation of cross-sectional malaria epidemiology studies because risk for transmission is highly dynamic and multifactorial. The longitudinal cohort data from both sites, once available, will allow for more precise understanding of the complex risk factors for incident malaria infection. The rich data sets will allow for more sophisticated analysis, for example, using complex mixed-effects regression models to account for repeated measures, clustering within household/village, and hierarchical levels of risk factors. Further, the identification and characterization of parasite populations will provide insights into complex malaria transmission dynamics in diverse scenarios, for example those characterized by changing seasonal endemic and/or epidemic transmission due to climate change, or those influenced by human mobility patterns due to social policies.

The high heterogeneity in malaria transmission in the Peruvian Amazon creates opportunities for targeted interventions [14]. However the identification of hotspots of malaria transmission and their characterization are not always straightforward. In our study, SaTScan analysis detected clusters with the highest malaria prevalence for both *P. vivax* and *P. falciparum* only in the CAH site. Since the cluster located on the south of CAH for *P. vivax* was found to be associated with an increased presence of male individuals, age groups older than 15 years and the lack of electricity at households, an adjusted spatial analysis for these covariates was performed resulting in the disappearance of the cluster. The observation indicating that the aggregation of *P. vivax* infections was mainly determined by the aggregation of groups with high-risk than by specific ecological conditions within the site has also been reported in other villages of the Peruvian Amazon [14]. Local mosquito and human mobility patterns may explain the absence of clustering of specific environmental factors along the Iquitos-Nauta road. Indeed, the presence of several breeding and resting sites near households along the road which enlarge after rains or floods from Itaya River could result into a wider dispersal of *An. darlingi*, and the high mobility of infected individuals through the unique and highly accessible road could further increase the dispersal of parasites. Conversely, the cluster for *P. falciparum* remained in the closest area to the river even after being adjusted for age and electricity availability (variables associated with *P. falciparum* infection). Its highest prevalence may be explained by the unprecedented floods from Itaya River in 2012 resulting in large and multiple breeding sites in the area, but also by the presence of a fluvial port to which individuals (resident and non-residents) arrive after being exposed to malaria due to travel-related economical activities in other endemic riverine villages.

## Conclusions

Parasitological measures (by LM and PCR), and individual and household characteristics collected during the baseline survey of a 3-year population-based malaria cohort study in the Amazonian Lupuna and Cahuide villages, together with contextual data obtained from malaria surveillance systems and health facilities, allowed for a good characterization of their population and malaria transmission patterns. On a population basis in both sites, low parasite rates were observed, with findings that asymptomatic and sub-patent infections predominated, compared to symptomatic and patent infections. However, the site-specific analysis of malaria prevalence and reported incidence showed substantial heterogeneity in malaria transmission at the micro-geographical level and over time, likely influenced by the site-specific socio-demographics, household characteristics, and ecological conditions in the villages. Riverine and road construction/deforestation contexts must be taken into account in order to carry out effective anti-malaria control and elimination efforts.

## Additional files

**Additional file 1: Table S1.** Baseline socio-demographic characteristics of study participants by villages. **Table S2.** Baseline household characteristics of study participants by villages.

**Additional file 2: Figure S1.** Weekly reported malaria cases in Cahuide (B) in 2012, and main control interventions implemented. (1) Mass screening and treatment (MS&T) and indoor spraying with residual insecticide with >90% of household coverage; (2, 3, 5, 6, 7) MS&T; (4) distribution of LLINs.
