# Supplementary material for: Micro-heterogeneity of malaria transmission in the Peruvian Amazon: a baseline assessment underlying a population-based cohort study
Source: Malar J. 2017 Aug 4;16:312. doi: 10.1186/s12936-017-1957-y (PMC5544973; doi:10.1186/s12936-017-1957-y)
Supplement: Supplementary file 1 — Additional file 1: Table S1. Baseline socio-demographic characteristics of study participants by villages. Table S2. Baseline household characteristics of study participants by villages. [file 12936_2017_1957_MOESM1_ESM.doc]

**Table S1**  Baseline socio-demographic characteristics of study participants by villages

|  |  | **LT** | | **SR** | | **SP** | | **CT** | | **LH** | | **DA** | |
| --- | --- | --- | --- | --- | --- | --- | --- | --- | --- | --- | --- | --- | --- |
|  |  | **n** | **%** | **n** | **%** | **n** | **%** | **n** | **%** | **n** | **%** | **n** | **%** |
| **Gender** | |  |  |  |  |  |  |  |  |  |  |  |  |
|  | Female | 171 | 48.6 | 152 | 52.1 | 83 | 44.9 | 334 | 45.5 | 69 | 46.9 | 107 | 46.3 |
|  | Male | 181 | 51.4 | 140 | 47.9 | 102 | 55.1 | 400 | 54.5 | 78 | 53.1 | 124 | 53.7 |
| **Age (years)** | |  |  |  |  |  |  |  |  |  |  |  |  |
|  | < 5 | 21 | 6.0 | 26 | 8.9 | 8 | 4.3 | 60 | 8.2 | 14 | 9.6 | 14 | 6.1 |
|  | 5-14.9 | 70 | 19.9 | 80 | 27.4 | 46 | 24.9 | 231 | 31.6 | 42 | 28.8 | 63 | 27.3 |
|  | 15-24.9 | 71 | 20.2 | 46 | 15.8 | 41 | 22.2 | 121 | 16.5 | 21 | 14.4 | 49 | 21.2 |
|  | 25-39.9 | 77 | 21.9 | 49 | 16.8 | 33 | 17.8 | 145 | 19.8 | 31 | 21.2 | 39 | 16.9 |
|  | 40-54.9 | 53 | 15.1 | 53 | 18.2 | 26 | 14.1 | 100 | 13.7 | 22 | 15.1 | 42 | 18.2 |
|  | > 55 | 60 | 17.0 | 38 | 13.0 | 31 | 16.8 | 75 | 10.2 | 16 | 11.0 | 24 | 10.4 |
| **Time in village (age≥10 years)** | | | |  |  |  |  |  |  |  |  |  |  |
|  | <2 | 13 | 4.5 | 8 | 3.6 | 5 | 3.2 | 158 | 29.1 | 13 | 11.9 | 36 | 18.8 |
|  | 2-9.9 | 16 | 5.5 | 30 | 13.6 | 28 | 18.2 | 204 | 37.6 | 41 | 37.6 | 43 | 22.5 |
|  | ≥10 | 263 | 90.1 | 183 | 82.8 | 121 | 78.6 | 181 | 33.3 | 55 | 50.5 | 112 | 58.6 |
| **Education (age≥18 years)** | | |  |  |  |  |  |  |  |  |  |  |  |
|  | None | 9 | 3.8 | 7 | 4.1 | 5 | 4.3 | 20 | 5.1 | 0 | 0.0 | 5 | 3.7 |
|  | Incomplete primary | 47 | 19.8 | 55 | 32.4 | 28 | 24.1 | 133 | 33.7 | 25 | 30.1 | 42 | 31.1 |
|  | Complete primary | 54 | 22.8 | 43 | 25.3 | 29 | 25.0 | 120 | 30.4 | 27 | 32.5 | 40 | 29.6 |
|  | Secondary | 112 | 47.3 | 61 | 35.9 | 53 | 45.7 | 115 | 29.1 | 28 | 33.7 | 42 | 31.1 |
|  | Superior | 15 | 6.3 | 4 | 2.4 | 1 | 0.9 | 7 | 1.8 | 3 | 3.6 | 6 | 4.4 |
| **Main occupation (age≥18 years)** | | | |  |  |  |  |  |  |  |  |  |  |
|  | None | 18 | 7.8 | 5 | 2.9 | 6 | 5.2 | 14 | 3.6 | 2 | 2.4 | 5 | 3.7 |
|  | Student | 10 | 4.3 | 8 | 4.7 | 6 | 5.2 | 6 | 1.5 | 1 | 1.2 | 2 | 1.5 |
|  | Housewife | 54 | 23.3 | 45 | 26.5 | 25 | 21.6 | 86 | 21.8 | 25 | 30.1 | 34 | 25.2 |
|  | Trader | 26 | 11.2 | 15 | 8.8 | 6 | 5.2 | 52 | 13.2 | 4 | 4.8 | 9 | 6.7 |
|  | Labourer,technic,professional | 38 | 16.4 | 7 | 4.1 | 27 | 23.3 | 70 | 17.8 | 6 | 7.2 | 12 | 8.9 |
|  | Guardian | 0 | 0.0 | 0 | 0.0 | 2 | 1.7 | 32 | 8.1 | 8 | 9.6 | 3 | 2.2 |
|  | Farmer | 78 | 33.6 | 62 | 36.5 | 38 | 32.8 | 104 | 26.4 | 32 | 38.6 | 44 | 32.6 |
|  | Logger | 6 | 2.6 | 12 | 7.1 | 4 | 3.4 | 24 | 6.1 | 5 | 6.0 | 26 | 19.3 |
|  | Fisher, hunter | 1 | 0.4 | 11 | 6.5 | 1 | 0.9 | 5 | 1.3 | 0 | 0.0 | 0 | 0.0 |
|  | Boat driver | 1 | 0.4 | 5 | 2.9 | 1 | 0.9 | 1 | 0.3 | 0 | 0.0 | 0 | 0.0 |
| **Lifetime malaria episodes** | | |  |  |  |  |  |  |  |  |  |  |  |
|  | 0 | 104 | 30.5 | 80 | 27.8 | 31 | 17.3 | 243 | 33.3 | 54 | 36.7 | 68 | 29.4 |
|  | 1 | 60 | 17.6 | 54 | 18.8 | 19 | 10.6 | 212 | 29.1 | 35 | 23.8 | 67 | 29.0 |
|  | 2-3 | 75 | 22.0 | 52 | 18.1 | 48 | 26.8 | 173 | 23.7 | 33 | 22.4 | 65 | 28.1 |
|  | ≥4 | 102 | 29.9 | 102 | 35.4 | 81 | 45.3 | 101 | 13.9 | 25 | 17.0 | 31 | 13.4 |
| **Malaria episodes (previous 12 months)** | | | | |  |  |  |  |  |  |  |  |  |
|  | 0 | 305 | 88.7 | 240 | 82.2 | 159 | 86.4 | 367 | 50.3 | 86 | 58.5 | 113 | 48.9 |
|  | 1 | 36 | 10.5 | 37 | 12.7 | 20 | 10.9 | 279 | 38.2 | 49 | 33.3 | 101 | 43.7 |
|  | 2-3 | 3 | 0.9 | 15 | 5.1 | 4 | 2.2 | 72 | 9.9 | 11 | 7.5 | 17 | 7.4 |
|  | ≥4 | 0 | 0.0 | 0 | 0.0 | 1 | 0.5 | 12 | 1.6 | 1 | 0.7 | 0 | 0.0 |

**Table S2. Baseline household characteristics of study participants by villages**

|  |  | **LT** | | **SR** | | **SP** | | **CT** | | **LH** | | **DA** | |
| --- | --- | --- | --- | --- | --- | --- | --- | --- | --- | --- | --- | --- | --- |
|  |  | **n** | **%** | **n** | **%** | **n** | **%** | **n** | **%** | **n** | **%** | **n** | **%** |
| **Overcrowding(>3 persons/bedroom)** | | | |  |  |  |  |  |  |  |  |  |  |
|  | No | 250 | 71.0 | 136 | 46.6 | 96 | 51.9 | 276 | 37.7 | 86 | 58.5 | 109 | 47.2 |
|  | Yes | 102 | 29.0 | 156 | 53.4 | 89 | 48.1 | 456 | 62.3 | 61 | 41.5 | 122 | 52.8 |
| **Wall material** | |  |  |  |  |  |  |  |  |  |  |  |  |
|  | Brick, cement | 123 | 34.9 | 7 | 2.4 | 0 | 0.0 | 49 | 6.7 | 2 | 1.4 | 0 | 0.0 |
|  | Wood | 194 | 55.1 | 264 | 90.4 | 171 | 92.4 | 565 | 77.2 | 145 | 98.6 | 209 | 90.5 |
|  | Palm | 23 | 6.5 | 21 | 7.2 | 3 | 1.6 | 39 | 5.3 | 0 | 0.0 | 22 | 9.5 |
|  | Tin, other | 12 | 3.4 | 0 | 0.0 | 11 | 5.9 | 79 | 10.8 | 0 | 0.0 | 0 | 0.0 |
| **Roof material** | |  |  |  |  |  |  |  |  |  |  |  |  |
|  | Tin | 200 | 56.8 | 65 | 22.3 | 6 | 3.2 | 107 | 14.7 | 19 | 12.9 | 31 | 13.4 |
|  | Palm | 152 | 43.2 | 227 | 77.7 | 179 | 96.8 | 619 | 85.3 | 128 | 87.1 | 200 | 86.6 |
| **Floor material** | |  |  |  |  |  |  |  |  |  |  |  |  |
|  | Cement | 157 | 44.6 | 33 | 11.3 | 13 | 7.0 | 106 | 14.5 | 14 | 9.5 | 21 | 9.1 |
|  | Wood | 9 | 2.6 | 21 | 7.2 | 68 | 36.8 | 362 | 49.5 | 126 | 85.7 | 152 | 65.8 |
|  | Dirt | 186 | 52.8 | 238 | 81.5 | 104 | 56.2 | 264 | 36.1 | 7 | 4.8 | 58 | 25.1 |
| **Electricity** | |  |  |  |  |  |  |  |  |  |  |  |  |
|  | Yes | 252 | 71.6 | 32 | 11.0 | 14 | 7.6 | 365 | 49.9 | 53 | 36.1 | 200 | 86.6 |
|  | No | 100 | 28.4 | 260 | 89.0 | 171 | 92.4 | 367 | 50.1 | 94 | 63.9 | 31 | 13.4 |
| **Potable water** | |  |  |  |  |  |  |  |  |  |  |  |  |
|  | Yes | 71 | 20.2 | 30 | 10.3 | 28 | 15.1 | 327 | 45.2 | 3 | 2.0 | 7 | 3.1 |
|  | No | 281 | 79.8 | 262 | 89.7 | 157 | 84.9 | 396 | 54.8 | 144 | 98.0 | 220 | 96.9 |
| **Source of water** | |  |  |  |  |  |  |  |  |  |  |  |  |
|  | Piped into dwelling | 0 | 0.0 | 0 | 0.0 | 6 | 3.2 | 39 | 5.7 | 5 | 3.4 | 0 | 0.0 |
|  | Public tap | 145 | 41.2 | 77 | 26.4 | 50 | 27.0 | 287 | 42.0 | 5 | 3.4 | 0 | 0.0 |
|  | Open well | 112 | 31.8 | 109 | 37.3 | 88 | 47.6 | 135 | 19.7 | 47 | 32.0 | 176 | 76.2 |
|  | River, rain | 95 | 27.0 | 106 | 36.3 | 41 | 22.2 | 223 | 32.6 | 90 | 61.2 | 55 | 23.8 |
| **Sanitation facility** | |  |  |  |  |  |  |  |  |  |  |  |  |
|  | Flush toilet | 4 | 1.1 | 0 | 0.0 | 0 | 0.0 | 0 | 0.0 | 3 | 2.0 | 0 | 0.0 |
|  | Pit latrine | 64 | 18.4 | 49 | 16.8 | 55 | 29.7 | 207 | 28.3 | 63 | 42.9 | 36 | 15.6 |
|  | Ground hole, cesspool | 68 | 19.5 | 122 | 41.9 | 89 | 48.1 | 346 | 47.3 | 43 | 29.3 | 129 | 55.8 |
|  | No facility, field | 212 | 60.9 | 120 | 41.2 | 41 | 22.2 | 179 | 24.5 | 38 | 25.9 | 66 | 28.6 |
| **Trash disposal** | |  |  |  |  |  |  |  |  |  |  |  |  |
|  | Burning trash | 114 | 32.4 | 42 | 14.4 | 84 | 45.4 | 309 | 42.2 | 95 | 64.6 | 63 | 28.4 |
|  | Bury trash | 24 | 6.8 | 16 | 5.5 | 16 | 8.6 | 97 | 13.3 | 18 | 12.2 | 48 | 21.6 |
|  | Field, river | 214 | 60.8 | 234 | 80.1 | 62 | 33.5 | 321 | 43.9 | 34 | 23.1 | 111 | 50.0 |
|  | Other | 0 | 0.0 | 0 | 0.0 | 23 | 12.4 | 5 | 0.7 | 0 | 0.0 | 0 | 0.0 |
| **Cooking fuel** | |  |  |  |  |  |  |  |  |  |  |  |  |
|  | Gas | 17 | 4.8 | 0 | 0.0 | 5 | 2.7 | 27 | 3.7 | 3 | 2.0 | 10 | 4.3 |
|  | Kerosene,charcoal | 10 | 2.8 | 3 | 1.0 | 0 | 0.0 | 78 | 10.7 | 5 | 3.4 | 54 | 23.4 |
|  | Firewood | 325 | 92.3 | 288 | 99.0 | 180 | 97.3 | 626 | 85.6 | 139 | 94.6 | 167 | 72.3 |
| **Bednet coverage (bednets/beds)** | | | |  |  |  |  |  |  |  |  |  |  |
|  | <80% | 0 | 0.0 | 23 | 7.9 | 5 | 2.7 | 19 | 2.6 | 0 | 0.0 | 13 | 5.6 |
|  | ≥80% | 352 | 100.0 | 269 | 92.1 | 180 | 97.3 | 713 | 97.4 | 147 | 100.0 | 218 | 94.4 |
| **Bednet material** | |  |  |  |  |  |  |  |  |  |  |  |  |
|  | None | 0 | 0.0 | 0 | 0.0 | 0 | 0.0 | 2 | 0.3 | 0 | 0.0 | 0 | 0.0 |
|  | Tocuyo | 19 | 5.4 | 10 | 3.4 | 12 | 6.5 | 297 | 40.6 | 26 | 17.7 | 77 | 33.3 |
|  | Nylon | 18 | 5.1 | 12 | 4.1 | 3 | 1.6 | 87 | 11.9 | 9 | 6.1 | 28 | 12.1 |
|  | LLINs | 315 | 89.5 | 270 | 92.5 | 170 | 91.9 | 332 | 45.4 | 112 | 76.2 | 113 | 48.9 |
|  | Other | 0 | 0.0 | 0 | 0.0 | 0 | 0.0 | 13 | 1.8 | 0 | 0.0 | 13 | 5.6 |
