# Supplementary figures and images for: Micro-heterogeneity of malaria transmission in the Peruvian Amazon: a baseline assessment underlying a population-based cohort study
Source: Malar J. 2017 Aug 4;16:312. doi: 10.1186/s12936-017-1957-y (PMC5544973; doi:10.1186/s12936-017-1957-y)

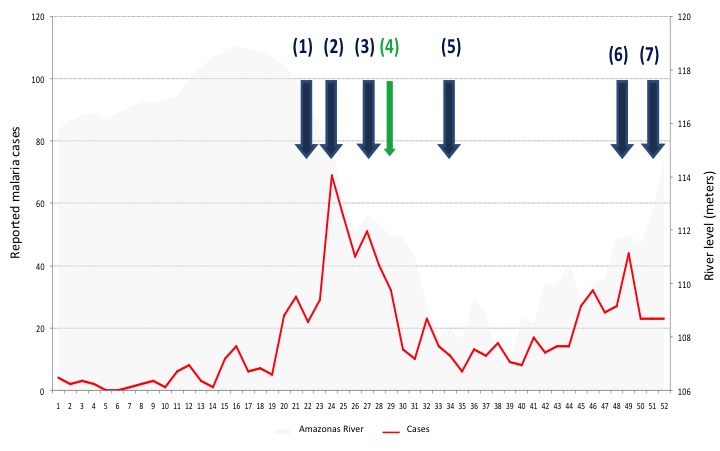

Supplement: Supplementary file 2 — Additional file 2: Figure S1. Weekly reported malaria cases in Cahuide (B) in 2012, and main control interventions implemented. (1) Mass screening and treatment (MS&T) and indoor spraying with residual insecticide with >90% of household coverage; (2, 3, 5, 6, 7) MS&T; (4) distribution of LLINs. [file 12936_2017_1957_MOESM2_ESM.jpg]
